# Activin A induces growth arrest through a SMAD- dependent pathway in hepatic progenitor cells

**DOI:** 10.1186/1478-811X-12-18

**Published:** 2014-03-17

**Authors:** Lin Chen, Wei Zhang, Hui-fang Liang, Qiao-dan Zhou, Ze-yang Ding, Hong-qiang Yang, Wei-bo Liu, Yan-hui Wu, Quan Man, Bi-xiang Zhang, Xiao-ping Chen

**Affiliations:** 1 Hepatic surgery centre, Tongji Medical College, Tongji Hospital, Huazhong University of Science and Technology, Wuhan, China; 2 Department of Nephrology, Tongji Medical College, Tongji Hospital, Huazhong University of Science and Technology, Wuhan, China; 3 Department of Hepatobiliary Surgery, First Affiliated Hospital, Medical College of Shihezi University, Shihezi, China

**Keywords:** Hepatic progenitor cell, Activin A, follistatin, Proliferation, Smads protein

## Abstract

**Background:**

Activin A, an important member of transforming growth factor-β superfamily, is reported to inhibit proliferation of mature hepatocyte. However, the effect of activin A on growth of hepatic progenitor cells is not fully understood. To that end, we attempted to evaluate the potential role of activin A in the regulation of hepatic progenitor cell proliferation.

**Results:**

Using the 2-acetaminofluorene/partial hepatectomy model, activin A expression decreased immediately after partial hepatectomy and then increased from the 9th to 15th day post surgery, which is associated with the attenuation of oval cell proliferation. Activin A inhibited oval cell line LE6 growth via activating the SMAD signaling pathway, which manifested as the phosphorylation of SMAD2/3, the inhibition of Rb phosphorylation, the suppression of cyclinD1 and cyclinE, and the promotion of p21^WAF1/Cip1^ and p15^INK4B^ expression. Treatment with activin A antagonist follistatin or blocking SMAD signaling could diminish the anti-proliferative effect of activin A. By contrast, inhibition of the MAPK pathway did not contribute to this effect. Antagonizing activin A activity by follistatin administration enhanced oval cell proliferation in the 2-acetylaminofluorene/partial hepatectomy model.

**Conclusion:**

Activin A, acting through the SMAD pathway, negatively regulates the proliferation of hepatic progenitor cells.

## Introduction

Oval cells are regarded as adult liver progenitor cells. They are able to differentiate either into mature hepatocytes or biliary epithelial cells [1]. These cells are termed as “oval cells” due to their characteristic morphology with an ovoid nucleus, small size (relative to hepatocytes) and high nuclear to cytoplasmic ratio [1,2]. In the 2-acetaminofluorene (2-AAF)/partial hepatectomy (PH) model, hepatocyte proliferation is efficiently suppressed by 2-AAF. This forces liver regeneration to become dependent on the replication and differentiation of oval cells [3,4]. After the restoration of the normal liver mass, any excess oval cells that have failed to differentiate into mature hepatocytes stop replicating and are eliminated through apoptosis to prevent liver hyperplasia [5]. The complex molecular events that trigger liver regeneration are now beginning to be elucidated [6], but little is known about the mechanisms that restrict proliferation and return oval cell to quiescence after liver regeneration.

Transforming growth factor-β1 (TGF-β1) has been proposed to negatively regulate the proliferation of hepatocytes [7] and oval cells [8-10] but its role is controversial. TGF-β1 over-expression results in impairment of oval cell expansion *in vivo*, inhibiting growth and inducing apoptosis in oval cell lines *in vitro*[10,11]. Conversely, other groups have demonstrated that TGF-β1 signaling is not necessary for restricting hepatocyte proliferation [12]. Furthermore, compared to hepatocytes, hepatic progenitor cells (HPCs) are more resistant to the anti-proliferative effects of TGF-β1 [9]. These data imply that there may be other factors that could recuperate the anti-proliferative effect of TGF-β1 in the regulation of oval cell liver regeneration.

Activin A, composed of two activin-βA subunits, is a member of the activin family, which in turn is part of the larger TGF-β superfamily. Activin A binds to its specific activin type II receptors (ActRIIA and ActRIIB), which leads to the recruitment and trans-phosphorylation of the partner activin type I receptor (ALK4). Activated ALK4 recruits and phosphorylates cytoplasmic receptor-regulated SMADs (R-SMAD), SMAD2 and SMAD3, which form a heterotrimer with SMAD4 (co-SMAD) and translocate into the nucleus where they control gene transcription [13]. Activin A has been shown to suppress proliferation and induce apoptosis of mature murine hepatocytes *in vivo* and *in vitro* by up-regulating p21^WAF1/Cip1^, p15^INK4B^ and down-regulating cyclin D1 and Cyclin-Dependent Kinase expression, and dephosphorylating Rb [14-18]. Moreover, intravenous or intraportal administration of follistatin, a specific antagonist of activin A, can accelerate liver regeneration in partially hepatectomized rats [14-17]. Never-the-less, the role of activin A in the regulation of hepatic oval cell proliferation has yet to be fully elucidated. In order to reveal the action and mechanism of activin A on hepatic oval cell proliferation, we first tested the expression pattern of activin A and follistatin in the 2-AAF/PH model. We then evaluated the response of a hepatic oval cell line to activin A *in vitro*. Finally, we blocked activin A activity by intra-portal administration of follistatin in the 2-AAF/PH model, and measured hepatic oval cell proliferation to demonstrate that activin A is an important negative regulator of hepatic oval cell mediated liver proliferation.

## Results

### Expression pattern of activin A and follistatin in 2-AAF/PH model

**Figure 1:**
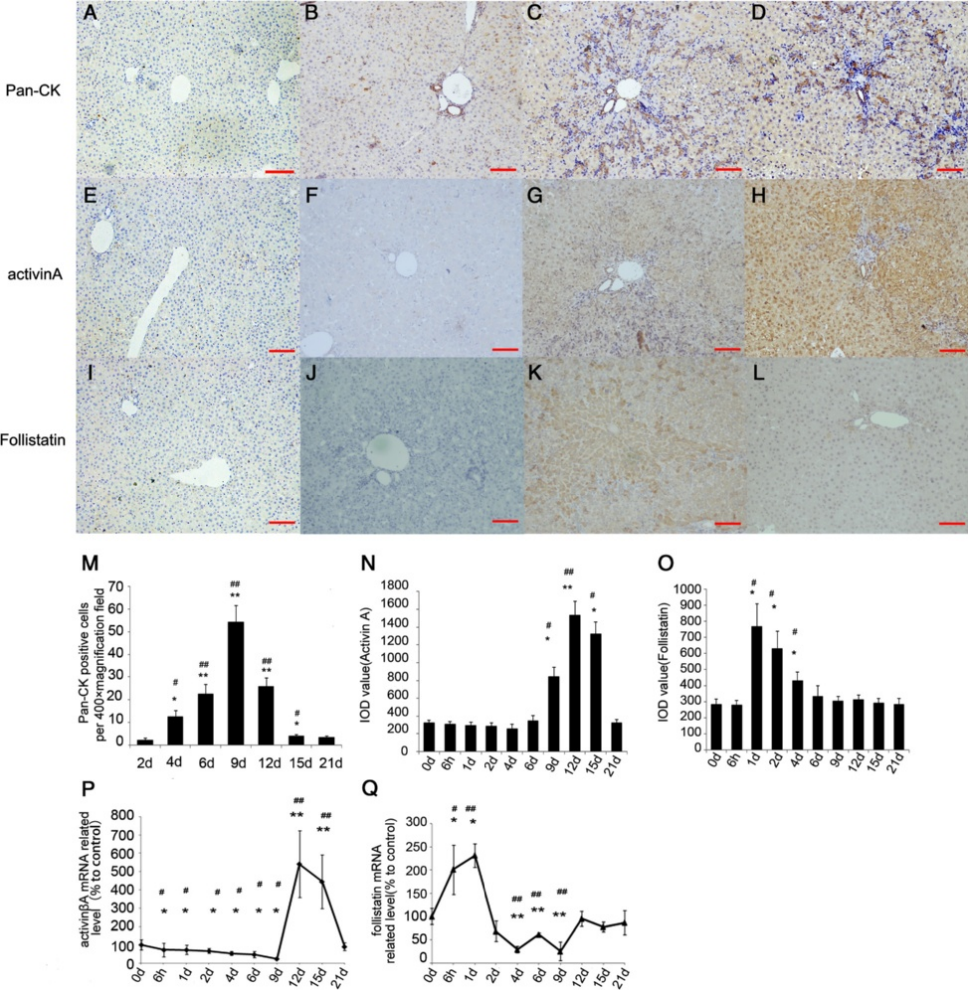
**Activin A and follistatin expression in the 2-AAF/PH model correlates with HPC proliferation. (A, E, I)** Negative control. **(B-D)** HPCs and interlobular or terminal duct cells showed positive immuno-reactivity (brown) to pan-CK monoclonal antibody. (×100 magnification). **(F-H)** Expression of Activin A in 2-AAF/PH model (×100 magnification). **(J-L)** Expression of Follistatin in 2-AAF/PH model (×100 magnification). **(B**, **F** and **J)** 2-AAF treated rats without PH; **(C, G)** 9th day after PH; **(D, H)** 15th day after PH; **(K)** 2th day after PH; **(L)** 4th day after PH. **(M)** The change of HPCs counts in residual liver tissue following PH in 2-AAF/PH model. Expression of activinA **(N)** and follistatin **(O)** were detected by IHC and IOD values were analyzed with Image-Pro Plus software (v. 5.0) and recorded in the histograms. **(P** and **Q)** Expression of activin βA **(P)** and follistatin **(Q)** mRNA in residual liver tissue of partial hepatectomic rats treated with 2-AAF. Data were log-transformed and analyzed by ANOVA for group difference (2-AAF/PH VS shame operation) and time difference (each time point VS 0 day) *P < 0.05 compared with initial time point (0 day). #*P* < 0.05 compared with shame operation group; **P < 0.01 compared with initial time point. ##*PP* < 0.01 compared with sham operation group. Bar = 100 um.

We evaluated the spreading of HPCs in 2-AAF/PH model using immuno-histochemical staining for Pan-Cytokeratin (Pan-CK), which is an established marker of HPC’s [18]. Recent studies have reported the activation of pan-CK positive HPCs in a number of HPC proliferation models including the 2-AAF/PH model [19-21]. In normal control and 2-AAF-treated sham operative rat liver, pan-CK mainly labeled interlobular and terminal duct cells. In the 2-AAF/PH model, high nuclei/cytoplasm ratio pan-CK positive cells (HPCs) initially appeared close to pre-existing bile ducts in or around portal tracts (Figure 1B). These cells became more numerous with time and reached a peak on the 9th day after PH (Figure 1C and M). From the 12th day to the 15th day post-surgery, the tubular structures and numbers of pan-CK positive cells dramatically decreased (Figure 1D and M), reaching a nadir between the 18th day to 21st day post-surgery (Figure 1M).

We next measured the expression of activin A and follistatin by immunohistology. Activin A expression showed no significant change until the 9th day, and then increased rapidly and reached a peak on the 12th day after surgery (Figure 1F to H and N). Follistatin level increased on the first day after surgery and was sustained until the 4th day (Figure 1J to L and O).

The transcript level of activin βA and follistatin mRNA did not vary dramatically in the sham operation group. Activin βA mRNA production decreased below baseline from 6 hours to 9 days following PH (*P* < 0.05), then increased from the 12th to 15th day after surgery, when the number of pan-CK positive HPCs began to decline (*P* < 0.01) (Figure 1P). Follistatin mRNA increased as soon as 6 hours after PH, (*P* < 0.05), and reached a peak at 24 hours (*P* < 0.05). After the peak, it declined below baseline from the 2nd day to 9th day after surgery (*P* < 0.01), then returned to the initial level on the 12th day (Figure 1Q). Activin βA mRNA expression decreased and its endogenous antagonist follistatin increased during the first phase of HPC dependent liver regeneration, while HPCs dramatically proliferated. At the end of the second week, the increase in activin βA expression coincided with the decline in HPCs. From these data, we presume that the activin A/follistatin axis may play an important role in the regulation of HPCs after partial hepatectamy.

### Activin A suppresses HPCs proliferation *in vitro*

**Figure 2:**
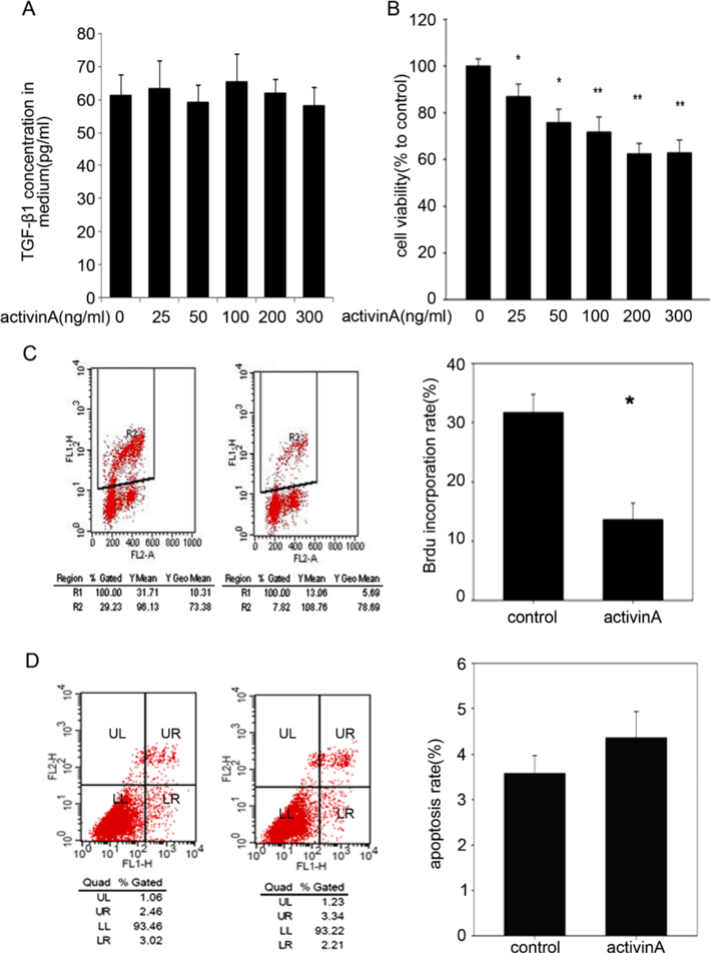
**Activin A inhibited proliferation of LE6 cells. (A)** 2 × 10^5^ LE6 cells/ were seed into 6-well plate and treated with serum free LE media in the present of activin A for up to 48 hours. Cell media was harvested and concentration of TGF-β1 was detected by ELISA kit. The figure showed result of one of three independent assays **(B)** LE6 cells were grown in 8% FBS LE media in the present or absence of activin A (25, 50, 100, 200, 300 ng/ml) for 72 hours. Cell proliferation was assessed by CCK-8 assay. Data showed result of one of three independent assays. **(C and D)** LE6 cells were grown in 8% FBS LE media in the presence or absence of activin A (200 ng/ml) for 72 hours. Cell DNA synthesis was assessed by BrdU incorporation assay using FACS **(C)**. R2 = BrdU positive cells. Cell apoptosis was assessed by AnnexinV/PI assay using FACS **(D)**. LL = Live cells or annexin V and PI negative cells, LR = Early apoptotic cells or annexin V positive cells, UR = Late apoptotic and necrotic cells or annexin V and PI positive cells, UL = Necrotic cells or PI positive cells. Bar chart showed the apoptosis rate (UR + LR) of control and activin treated LE6 cells. The figure showed result of one of three independent assays.

In order to confirm our hypothesis, we tested the effect of exogenous activin A on cells from the rat HPC cell line LE6. First, we examined the baseline of endogenous activin A and TGF-β1 in LE6 cells by enzyme-linked immunosorbent assay (ELISA). Any activin A secreted by LE6 cell cultures was below the minimum detectable dose of the ELISA kit (data not shown). LE6 cells secreted detectable quantities of TGF-β1 which amounted to 60 pg/ml in the culture supernatants, and the addition of exogenous activin A to the cell cultures did not alter their expression of either activin A or TGF-β1 (Figure 2A). Therefore, we could ignore the interference of endogenous activin A and TGF-β1. Next, we treated LE6 cells with various doses of exogenous activin A and measured the effect of this cytokine on LE6 cell proliferation. As cell counting kit-8 (CCK-8) assay results showed in Figure 2B, activin A significantly inhibited proliferation of LE6 cells in a dose-dependent manner. Over a 72 h incubation period, 25 ng/ml activin A was sufficient to induce growth inhibition in LE6 cells. A maximum of 40% inhibition of proliferation was achieved with 200 ng/ml activin A. Accordingly, BrdU label index assay showed 200 ng/ml activin A inhibited as much as about 50% DNA synthesis in LE6 cells (Figure 2C). We next explored the effect of activin treatment on induction of apoptosis in LE6 cells using an AnnexinV/PI double label assay. As seen in Figure 2D, 200 ng/ml activin A barely induced apoptosis in LE6 cells (Figure 2D).

### Rb protein, cyclinD1, cyclinE, p15^INK4B^ and p21^WAF1/Cip1^ are involved in activin A induced growth arrest in LE6 cells

**Figure 3:**
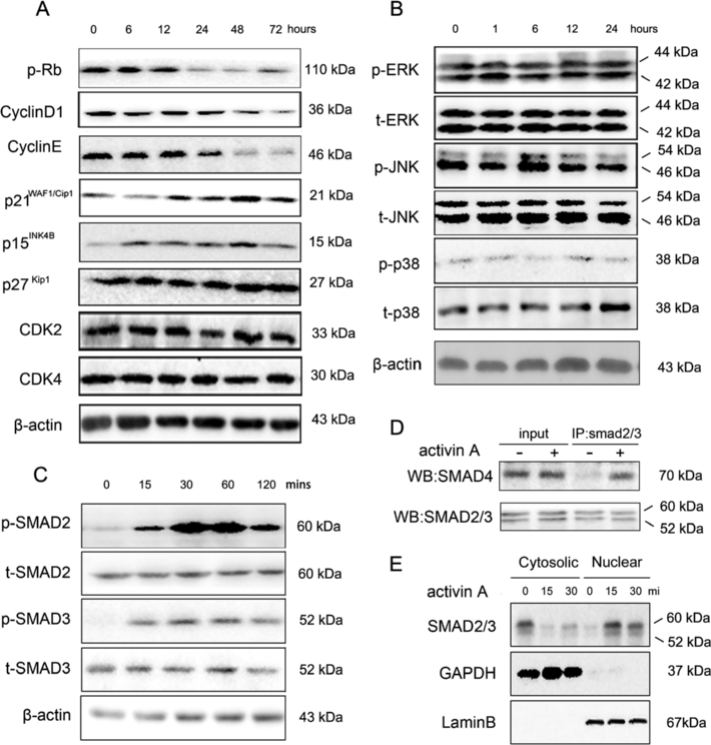
**Activin A activates SMAD signaling in LE6 cells. (A)** LE6 cells were treated with activin A (200 ng/ml) for 6 h, 12 h, 24 h 48 h and 72 h, cell lysis was analyzed by western-blot using specific antibody to phosphorylated Rb, cyclinD1, cyclinE , p21^WAF1/Cip1^, p15^INK4B^ p27^Kip1^, CDK2 and CDK4. β-actin was used as a loading control. **(B)** LE6 cells were treated with activin A (200 ng/ml) for 1 h, 6 h, 12 h and 24 h, cell lysis was analyzed by western-blot using specific antibody to phosphorylated JNK1/2, ERK1/2 and p38. **(C)** LE6 cells were treated with activin A (200 ng/ml) for 15 min, 30 min, 60 min and 120 min, cell lysis was analyzed by western-blot using specific antibody to phosphorylated SMAD2, SMAD3. **(D)** LE6 cells were treated with activin A (200 ng/ml) for 60 min, cell lysis was incubated with anti-SMAD2/3 antibody and protein A/G agarose overnight, then analyzed by western-blot using specific antibody to SMAD4. **(E)** LE6 cells were treated with activin A (200 ng/ml) for 15 min and 30 min, Nuclear and cytosolic fractions of cells were analyzed by western-blot using specific antibody to SMAD2/3.GAPDH or laminB was used as loading control for cytosolic or nuclear fraction.

To investigate the mechanism underling activin A-induced growth arrest in LE6 cells, we treated LE6 cells with activin A and detected a series of cell cycle related proteins by western-blot. As shown in Figure 3A, the protein expression of the cyclin dependent kinase inhibitors p15^INK4B^ and p21^WAF1/Cip1^ increased dramatically after 6 h and 12 h respectively, and in both cases this was sustained until 72 h after activin A stimulation. Conversely, cyclin D1 and cyclin E expression decreased significantly 24 hours after exposure to activin A. Moreover, phosphorylation of Rb was also inhibited by activin A. By contrast, activin A stimulation was unable to affect the extent of p27^Kip^, cyclin-dependent kinase 2 (CDK2) and cyclin-dependent kinase 4 (CDK4) expression in LE6 cells. These results indicated that activin A suppressed downstream targets cyclin D1 and cyclin E, induced expression of p21^WAF1/Cip1^ and p15^INK4B^, and dephosphorylated Rb protein all of which are likely to contribute to cell growth arrest.

### Activin A failed to activate SMAD-independent mitogen-activated protein kinase signaling in LE6 cells

Next we investigated the signaling pathways, activated by activin A, that are required for growth arrest. The principal pathways include the SMAD and mitogen-activated protein kinase (MAPK) signaling cascades [22]. First, we investigated the MAPK pathway by measuring phosphorylated ERK1/2, JNK1/2 and p38MAPK in LE6 cells by western-blot (Figure 3B). In the absence of activin A stimulation, all three kinases were constitutively phosphorylated in LE6 cells, and their phosphorylation was not affected by activinA treatment. These data suggest that MAPK signaling did not contribute to activinA induced growth arrest in LE6 cells.

### SMAD dependent signaling is necessary for activin A induced growth arrest in HPCs

Next we looked at the SMAD pathway. LE6 cells were incubated with the indicated dose of activin A, and then phosphorylated SMAD2 and SMAD3 were analyzed by western-blot. The data showed that SMAD2 and SMAD3 were phosphorylated in a time-dependent manner (Figure 3C). Phosphorylated SMAD2/3 forms a complex with SMAD4 that shuttles into nucleus where they regulate downstream gene transcription [22]. We detected the formation and location of SMAD2/3/4 heterotrimer in activin A treated LE6 cells. The co-immunoprecipitation (co-IP) results demonstrated activin A induced SMAD2/3/4 complex formation in LE6 cells (Figure 3D) and western-blot results indicated that SMAD2/3 was predominantly located in nucleus in activin A treated LE6 cells, while conversely, SMAD2/3 was principally located in cytoplasm in control cells (Figure 3E). These data confirm that activin A is able to activate the canonical SMAD signaling pathway in LE6 cells.

### SMAD4 knockdown interrupts activin A-induced growth arrest in LE6 cells

**Figure 4:**
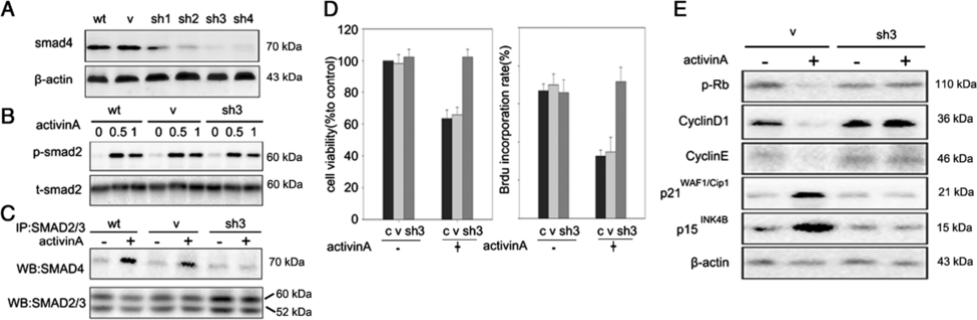
**Knockdown of *Smad4 *blocks the anti-proliferative effect of activin A in LE6 cells. (A)** SMAD4 expression was determined by western-blot in LE6 cells after *Smad4* knockdown (sh1, 2, 3, 4), and compared with control cells (wt) and vehicle cells (V). **(B and C)** Control cells, vehicle and *Smad4* knockdown LE6 cells (LE6-*Smad4*KD) were treated with or without 200 ng/ml activin A. Phosphorylated SMAD2 was detected by western-blot **(B)** and SMAD2/3/4 complex formation was detected by co-immuno precipitation **(C)**. **(D)** sh*Smad4*-LE6 cells were treated with or without 200 ng/ml activin A and their proliferation assessed by CCK-8 and BrdU incorporation assay. c: control, v: vehicle, sh3: LE6-smad4KD. **(E)** Indicated cells were treated with or without 200 ng/ml activin A, phosphorylated Rb, cyclinD1, cyclinE , p21^WAF1/Cip1^and p15^INK4B^ were analyzed by western blotting.

SMAD4 is the pivotal factor of canonical SMAD signaling and its inactivation or deletion prevents SMAD signaling. To further investigate the role of the SMAD pathway in activin A-mediated growth arrest, LE6 cells were infected with LV-*shSmad4* to stable knockdown endogenous *Smad4*. 3 of 4 *Smad4* shRNA oligonucleoties were able to deplete *Smad4* expression by more than 70% in LE6 cells and we chose the most effective sequence sh3 for the following study (Figure 4A). Activin A stimulated SMAD2 and SMAD3 phosphorylation (Figure 4B) but failed to induce formation of functional SMAD2/3/4 heterotrimer in *Smad4* knockdown LE6 cells (LE6-*Smad4*KD) (Figure 4C). These data indicated that activin A-induced SMAD signaling could be blocked by *Smad4* knockdown.

We next explored the effect of depleting SMAD4 on the ability of activin A to induce a growth arrest. LE6 cells transferred with an empty vector remained sensitive to the effects of activin A, whereas LE6-*Smad4*KD were resistant to activin A induced growth inhibition. Then we examined the effect of activin A on the target protein expression in LE6-*Smad4*KD cells (Figure 4E). As expected, activin A induced expression of p21^WAF1/Cip1^and p15^INK4B^ in LE6-wild type (LE6-WT) cells, but it could not induce these proteins in LE6-*Smad4*KD cells. Consistent with this, activin A failed to down-regulate cyclin E, or cyclin D1, or phosphorylated Rb in LE6-*Smad4*KD cells. These results confirmed that SMAD4-dependent signaling was crucially involved in activin A induced growth inhibition in HPCs.

### Follistatin antagonizes activin A induced growth arrest in HPCs

**Figure 5:**
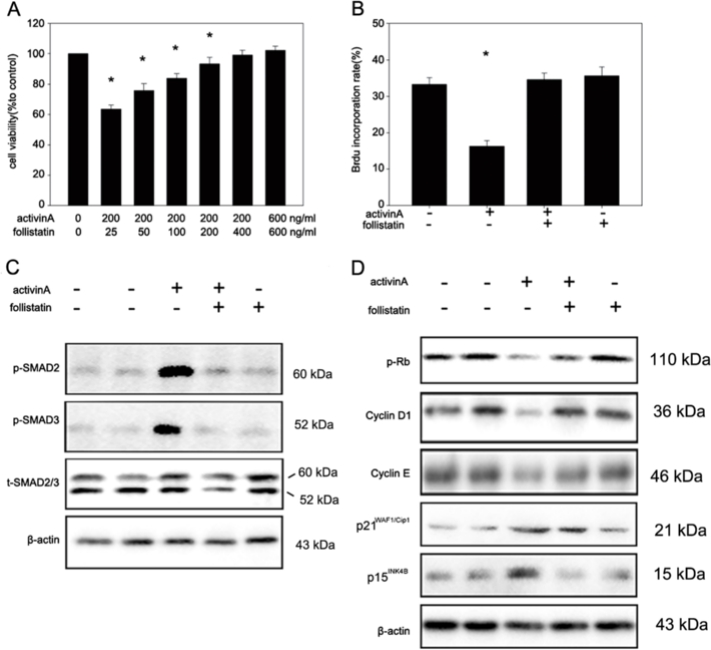
**Follistatin blocks the growth arrest induced by activin A in LE6 cells. (A)** LE6 cells were treated with activin A (200 ng/ml) in the presence or absence of the indicated doses of follistatin (25, 50, 100, 200, 400, 600 ng/ml). Cell proliferation was detected by CCK-8 assay. **(B)** LE6 cells were grown in LE media in the presence or absence of activin A (200 ng/ml), follistatin (400 ng/ml) or activin A (200 ng/ml) plus follistatin (400 ng/ml). DNA synthesis was detected by BrdU incorporation assay using FACS. **(C)** LE6 cells were treated with or without activin A (200 ng/ml), follistatin (400 ng/ml) or activin A (200 ng/ml) plus follistatin (400 ng/ml) for 30 min. Phosphorylated SMAD2/3 was detected by western-blot. **(D)** LE6 cells were treated with either media alone, activin A (200 ng/ml), follistatin (400 ng/ml) or activin(200 ng/ml) plus follistatin (400 ng/ml). Then phosphorylated Rb, cyclinD1, cyclinE, p21^WAF1/Cip1^and p15^INK4B^ were analyzed by western-blot.

We found that follistatin mRNA increased in the early phase of HPC-mediated liver regeneration, which was about 6 h after PH and was followed by HPC proliferation. These data indicated that follistatin could interrupt the tonic growth inhibitory effect of activin A and in turn stimulate HPC-induced liver regeneration. To confirm this hypothesis, we treated LE6 cells with either activin A, or activin A together with increasing-doses of follistatin or follistatin alone, then analyzed the proliferation using CCK-8 and BrdU incorporation assay. As seen in Figure 5A and B, 400 ng/ml follistatin could fully reverse 200 ng/ml activin A-induced growth arrest in LE6 cells. Nevertheless, follistatin alone was unable to regulate cell proliferation. Yet, follistatin therapy completely inhibited activin A-induced phosphorylation of SMAD2 and SMAD3, restored expression of cyclin D1 and cyclin E suppressed by activin A and suppressed p21^WAF1/Cip1^ and p15^INK4B^ expression induced by activin A (Figure 5C and 5D). These data indicated that follistatin could inhibit activin A induced growth arrest.

### Follistatin boosts HPC proliferation *in vivo*

**Figure 6:**
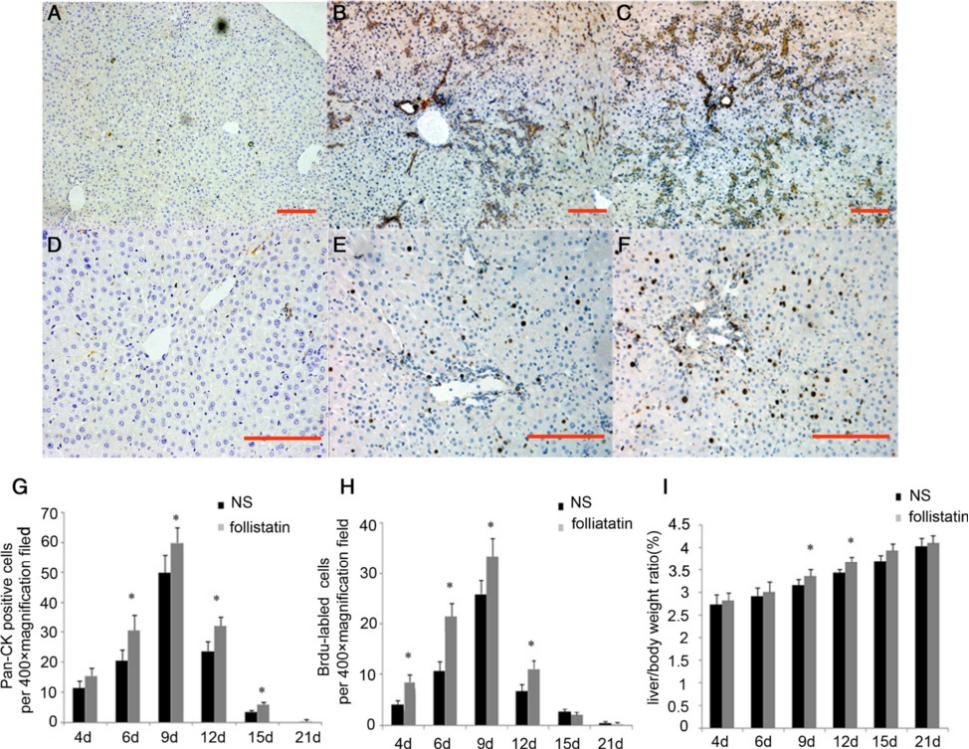
**Follistatin accelerated oval cell proliferation *in vivo*. (A and D)** negative control; Pan-CK positive hepatic progenitor cells (**B** and **C**, 100 × magnification) and BrdU positive proliferating cells (**E** and **F**, 200 × magnification) in normal sailine (NS) or follistatin treated (FST) 2-AAF/PH rats 6 day after PH were determined by immuno-histochemistry. Comparison of the number of Pan-CK positive hepatic progenitor cells **(G)**, BrdU positive proliferating cells **(H)** and Liver /body weight ratio **(I)** in two groups of animals. Data represent mean ± SD, n = 4–6, *P < 0.05 compared to NS group. Bar = 100 um.

In order to confirm the anti-proliferation role of activin A *in vivo*, follistatin or normal saline (NS group) was infused into portal vein immediately after 70% PH and into the tail vein at 5, 10, 15 and 20 days after PH in 2-AAF/PH rats. Compared to the NS group, more Pan-CK positive hepatic progenitor cells were present in follistatin-treated rats at 6, 9, 12, 15 days after PH. However, no significant differences were detected in rats at 4 days and 21 days after PH (Figure 6B, 6C and 6G). Next we detected the proliferation of cells in both groups by BrdU label assay. More BrdU-positive cells were detected in the follistatin-treated group at 4, 9, 12 days after PH. In addition, there were no significant differences between these 2 groups at 15 and 21 days after PH (Figure 6E, 6F and 6H). To exclude potential errors from body weight variations, liver/body weight ratios were used to assess remnant liver restoration. The ratio in the follistatin treated group was dramatically higher than NS group at 9 days, 12 days and 15 days after PH (Figure 6I). These data indicate that follistatin, an activin A antagonist, could enhance and prolong hepatic progenitor cell amplification *in vivo*. These results confirmed the anti-proliferation effect of activin A on hepatic progenitor cells *in vivo*.

## Discussion

In the present study, we examined the inhibitory effect of activin A on the proliferation of hepatic progenitor cells and revealed the mechanism. Activin A has been previously reported to inhibit DNA synthesis in mature hepatocytes [12,15-17,23,24]. After 2/3 PH, activin A expression is decreased and follistatin is induced during the growth period, whereas activin A expression is significantly induced in the later stages when regeneration is slowing down [25]. We observed a similar behavior in the 2-AAF/PH HPC-mediated liver regeneration model. Furthermore, DNA synthesis was enhanced in intact rat liver in which activin A signaling was temporary disrupted by administration of follistatin [26-28]. Our data demonstrated follistatin enhanced proliferation of HPCs in the 2-AAF/PH model. All these findings indicate that a basal amount of activin A exists in the intact liver to tonically inhibit mature hepatocyte or HPC-mediated proliferation and maintained adequate liver weight. Moreover, it plays an important role in termination of liver regeneration. Once the balance between follistatin and activin A is broken, for example, in the PH or 2-AAF/PH model, hepatocyte or hepatic progenitor cells escape from the anti-proliferative effect of activin A and start to proliferate. Once the liver mass is restored, the restoration of activin A expression again terminates liver regeneration, irrespective of whether the regeneration is mediated by hepatocytes or hepatic progenitor cells.

Ooe *et al.*[29] described a subpopulation of rat hepatocytes (small hepatocyte, SH) that have high growth potential in culture. Recent studies indicated that SH are posterity of oval cells: Ichinohe and Xiang reported that oval cells could differentiate into mature hepatocyte via SH [30-32]. Consistent with our work, Ooe found activin A suppresses the proliferation of SH, but cannot induce apoptosis of SH. Both studies demonstrate that the activin pathway is a key negative regulator of hepatic progenitor proliferation. However, Ooe reported activin B suppressed the proliferation of SH in an autocrine manner. The contribution of activin B and other members of the activin family to the proliferation of oval cells needs further investigation. Menthena *et al.*[33] reported fetal liver progenitor cells were resistant to activin A because of their lack of activin A receptors. The inconsistency between both Menthena and Ooe’s studies and our results may be due to different activin A receptor expression. In our hands and in Ooe’s work activin receptors are abundantly expressed in adult hepatic progenitor cells. This fact suggests that there are major differences in the regulation of cell growth between adult and fetal liver progenitor cells.

The anti-proliferative effects of activin A are likely to be due to the activation of signaling pathways that target cell cycle-related proteins. Activin A enhanced expression of p15^INK4B^, reduced cyclin A expression and reduced phoshorylation of the Rb protein in breast cancer cells [34]. Hepatoma cells respond to activin by up-regulating the expression of p21^WAF1/Cip1^, p16 and p15^INK4B^ proteins that suppress the cyclinD-CDK4/6 and cyclinE-CDK2-mediated phosphorylation of the Rb protein [23,35,36]. Activin A could down-regulate cyclin D, cyclin E and CDK4, all of which are important contributors to Rb protein phosphorylation [34,36]. The dephosphorylation of Rb leads to cell cycle arrest and inhibtion of cell proliferation. Our study demonstrated that activin A stimulated the expression of p15^INK4B^ and p21^WAF1/Cip1^ and inhibited the expression of cyclin D1 and cyclin E protein leading to the inhibition of Rb protein phosphorylation. These data indicated that p15^INK4B^, p21^WAF1/Cip1^, cyclin D1 and cyclin E were all associated with regulating the degree of Rb protein phosphorylation during activin A-induced cell proliferation arrest in HPCs.

Activin A regulates cell proliferation in numerous types of cells via SMAD signaling. The activation of the activin A/SMAD pathway results in the formation and nuclear location of the SMAD2/3/4 complex and regulates the expression of known targets including c-myc, cdc25A, p15^INK4B^, p21^WAF1/Cip1^, p16 ^INK4A^, cyclinA, cyclinD1 and cyclinE [37-39]. However, other studies reported that not only SMAD, but also p38MAPK and ERK signaling contribute to activin A-induced proliferation arrest or apoptosis [40,41]. Our study confirmed that activin A activated SMAD pathway (SMAD2/3 phosphorylation, SMAD2/3/4 complex formation and nuclear location), and regulated downstream targets expression (p15^INK4B^, p21^WAF1/Cip1^, cyclin D1 and cyclin E) in HPCs. Destruction of SMAD signaling by SMAD4 knockdown fully restrained activin A-induced proliferation arrest in LE6 cells. Moreover, activin A failed to change the phosphorylation level of p38 and ERK in LE6 cells. These data indicate that the anti-proliferation effect of activin A is SMAD-dependent. Nevertheless, we noticed a high basal level of phosphorylated p38, ERK and JNK in serum-starved LE6 cells, which might be related to the autocrine production of growth/survival factors, such as hepatocyte growth factor (HGF) and epidermal growth factor (EGF). These autocrine signals may be responsible for the insensitivity of MAPK pathways to respond to the addition of exogenous activin A. Furthermore, the over-activation of MAPK might be also responsible for our observation that LE6 cells were more insensitive to activin A-induced growth arrest and apoptosis compared to previously reported studies in mature hepatocytes [35,42].

The biological function of follistatin has been based on its reported ability to bind to activins with a high affinity. The picomolar affinity of follistatin molecules for activin dimmers forms the basis for follistatin to act as a potent extracellular regulatory mechanism in which activins are tightly bound and cannot bind to activin receptors and trigger downstream signaling [43]. Ooe *et al.* reported follistatin facilitates the proliferation of small hepatocytes by blocking activin A signaling in an autocrine manner. Administration of follisatin accelerated proliferation of hepatocyte growth *in vivo*. In the 2-AAF/PH model, up-regulation of follistatin in rat livers decreased the activity of activin A signaling and rendered cells resistant to activin A-induced growth arrest. Administration of follistatin accelerated oval cell expansion in the 2-AAF/PH model. Yet, follistatin alone was unable to affect the proliferation of LE6 cell. Taken together, our data indicated follistatin regulated oval cell proliferation only by blocking activin A. Our data also indicated that although follistatin itself did not have the ability to work as mitogen, it could neutralize the growth arrest of activin A and facilitated the proliferation of hepatic progenitor cells.

In conclusion, our study showed the compact correlation between activin A signaling and HPC proliferation. Furthermore, we found activin A inhibited cellular proliferation in HPC cell lines via the canonical SMAD pathway. Activin A up-regulated p15^INK4B^ and p21^WAF1/Cip1^, down-regulated cyclin D1 and cyclin E. Consistent with our results, it is reported that reduced phosphorylation of Rb protein, is associated with a growth arrest in HPCs. Taken together, activin A plays an important role in negative regulation of HPCs proliferation through a SMAD-dependent pathway.

## Material and methods

### Animal model

Adult male Sprague–Dawley rats (180-200 g) were used. They were bred and maintained on standard laboratory chow using 12-hour light/dark cycles. The body weights were recorded daily. The rats were treated according to the guidelines of the council for International Organizations of Medical Sciences, as required by the ethics committee of Tongji Medical College.

The rats 2-AAF/PH model were made as previously described [44]. Briefly, all rats received oral gavages of 2-AAF (Sigma-Aldrich MO USA) dissolved in polyethylene glycol (mol. wt. 400, Sigma-Aldrich MO USA) for up to 11 days at the dose of 15 mg/kg, and then two-thirds PH was performed under pentobarbitone anaesthesia in the fifth day. Control sham operation consisted of laparotomy without PH and briefly handling the liver. Five rats were killed at 6 h, 1d, 2d, 4d, 6d, 9d, 12d, 15d and 21d after initiation of PH. Liver tissue samples were fixed in 4% paraformaldehyde and processed to 4 μm thick sections for immunohistochemistry or immediately stored in −80°C for real-time PCR.

### Follistatin administration

After 70% PH, 1microgram follistatin dissolved in 0.5 ml natural saline was infused into portal vein using 29G insulin syringe as mentioned by Kogure [14]. In the normal saline (NS) group, the same volume of normal saline was infused into portal vein. The same dose of follistatin or saline was injected into tail vein at 5, 10, 15 and 20 days after PH.4-6 rats were killed at 4d, 6d, 9d, 12d, 15d, 21d after initiation of PH, liver weights and body weights were recorded. Restoration of liver weights was expressed as percentage of regenerated liver weight to body weight. For BrdU incorporation assay, two hours before sacrifice, animals were injected intraperitoneally with 50 mg 5-bromo-2-deoxyuridine (BrdU) per kg body weight. Liver tissue samples were fixed in 4% paraformaldehyde for immunohistochemistry.

### Immunohistochemistry

Sections were deparaffinised as described previously. Antigens were retrieved by incubating with proteinase K (DakoCytomation, Glostrup, Denmark) at 4°C for 5 min or by undergoing microwave heat antigen retrieval in 10 mM Tris Base, 1 mM EDTA Solution, pH 9.0; For BrdU incorporation assay, sections were incubated in 4 N HCL for 20 min at 37°C, after rinsing in 0.1 M borax solution for 5 min and PBS for 3 × 5 min, sections were incubated with 0.1% trypsin for 10 min at 37°C. Endogenous peroxidase activity was blocked with 3% H_2_O_2_ in methanol. Then sections were incubated with rabbit anti-pan-cytokeratin antibody (1:50, DAKO, Denmark), mouse anti-activin A antibody (1:100, R&D, USA), rabbit anti-follistatin antibody (1:50, ProteinTech Group, China) or mouse anti-BrdU antibody (1:400, DAKO Denmark) at 4°C overnight. For negative control, antibodies were replaced by homologous serum. Sectionswere washed with PBS and subsequently incubated with goat anti-mouse EnVision kit (DAKO Denmark) at room temperature. Peroxidase activity was detected using 3'3'diaminobenzidine tetrahydrochloride (DAB, DAKO Denmark) and counterstained with haematoxylin (Sigma-Aldrich MO USA). Digital images were prepared by Digital Sight ACT-1 for L-1 Software (Nikon Japan). Positively stained cells were counted as described previously with slight changes [30,45]. In brief, BrdU positive cells and hepatic progenitor cells positive for Pan-CK were counted in 20 adjacent non-overlapping fields in one section, at 400× magnification. 5 non-serial sections were counted for each rat. The expression of follistatin and activinA were annualized as described previously [46]. In brief, average value of IOD was obtained by analyzing five fields at 200× magnification per slide with Image-Pro Plus software (V.5.0).

### Real-time PCR

Total RNA was extracted from liver tissue sample using Trizol regent (Invitrogen, CA USA) according to manufacturer’s directions. Equal amounts of RNA were used for generation of first strand cDNA using PrimeScript® RT reagent Kit (Takara Bio Inc, Japan). Primers used to detect activin βA [Gene Bank: NM_017128.2], follistatin [Gene Bank: NM_012561.1] and β-actin were designed using the primer design software ‘Primer 5.0’ (Table 1) and purchase from suppliers (Invitrogen, CA USA). Real-Time PCR was performed on ABI Prism 7000 Sequence Detection System with ABI Prism 7000 SDS Software 1.0 in 96-well format and 25 μL reaction volume per well (Applied Biosystems, CA USA) with SYBR Green Real-time PCR Master Mix-PLUS (TOYOBO, Japan) according to the manufacturers’ introductions. The Ct values of the selected genes were first normalized with β-actin from the same sample, and then the relative expression of each gene was analyzed using 2^-ΔΔCt^ Method.

**Table 1:** The primer sequences and melting temperature(Tm) of the examined genes

**Gene**	**Sense**	**Antisense**	**Tm°C**
Activin βA	5'-TGATGTGCGGATTGCTTGTG-3'	5'-ACTGCGGTGAGGATGGTCTT-3'	60
Follistatin	5'-AGCTTGCTGGGCAGATCCATT-3'	5'- TACAGACGGGCTCATCCGACTT-3'	60

### Cell culture

Hepatic progenitor cell line LE6, derived from rats that were maintained on a choline-deficient diet for 6 weeks [47-49], were a kind gift from Prof. Nelson Fausto. Cells were grown in DMEM/F10 (Thermo Scientific HyClone, Utah USA) supplemented with 10% fetal bovine serum (FBS, Thermo Scientific HyClone, Utah USA), 0.5 mg/L insulin, 1 mg/L hydrocortisone (Sigma-Aldrich, MO USA) and 50 mg/L gentamicin(Invitrogen, CA USA) (LE medium) and were cultured in 37°C and 5% carbon dioxide.

### Lentivirus transfection of LE6 Cells

Rat *Smad4*-specific shRNA lentivirus and random shRNA lentivirus were purchased from GeneChem Co Shanghai China (Table 2) [Gene Bank: NM_019275]. LE6 cells were incubated with *Smad4*-specific shRNA lentivirus particles at a ratio of 25 particles to 1 cell, in the presence of 8 μg/ml hexadimethrine bromide to improve transduction efficiency. Random oligo shRNA lentivirus was used as control. Stable *Smad4* knock down LE6 cells were selected by 2ug/ml puromycin. *Smad4* knockdown effects from four shRNA sequences were detected by western-blot and the most efficient sequence was used for further research.

**Table 2:** Target sequences of specific shRNA oligoes to rat smad4

**Marker**	**Gene**	**Target sequence**	**GC%**
Sh1	Smad4	GCTACTTACCACCATAACA	42.10%
Sh2	Smad4	GGTAGGAGAGACATTTAAA	36.84%
Sh3	Smad4	GGAGTGCAGTTGGAGTGTA	52.63%
Sh4	Smad4	GCTGAAAGAGAAGAAAGAT	36.84%

### Activin A and TGF-β1 enzyme-linked immunosorbent assay

Supernatants were collected from confluent LE6 cells treated with or without activin A and tested in triplicate for activin A and TGF-β1 concentrations using rat activin A ELISA kit and rat TGF-β1 ELISA kit (R&D system, MN USA). 3 independent assays were performed with at least 3 replicates.

### Proliferation and apoptosis assay

Growth property of LE6 cells was tested by CCK-8 assay according to manufacturer’s introductions (Beyotime Institute of Biotechnology, China). Briefly, LE6 cells were seeded in triplicates in 96-well plate at 800 cells/100ul LE medium. Cells were either stimulated with or without various concentrations of activin A (PeproTech Inc, NJ USA), follistatin (R&D system, MN USA) or activin A(200 ng/ml) plus follistatin (400 ng/ml). After 72 hours incubation at 37°C, cell viability was determined by colorimetric assay using CCK-8. 3 independent assays were performed with at least 3 replicates.

For BrdU incorporation assay, LE6 cells were planted in triplicate in 6-well-plate at 1.5 × 105 cells/well. Cells were incubated with activin A (200 ng/ml), follistatin (400 ng/ml) or activin A (200 ng/ml) plus follistatin (400 ng/ml) for 72 hours and 10uM BrdU (Sigma-Aldrich, MO USA) were added at the last 30 min. Cells were harvested and fixed by ice cold 70% ethanol. After treated with 4 N HCL and 0.2 M borax, cells were incubated with mouse anti-BrdU monoclonal antibody (1:100, Santa Cruz Biotechnology, CA USA) for 1 hour at 4°C and FITC labeled goat anti-mouse antibody (1:100,Jackson ImmunoResearch Laboratories Inc, PA USA) for 1 hour at 37°C. Then BrdU incorporation rate was examined by BD FACSCanto™ Flow Cytometry System (Becton, Dickinson and Company, NJ USA). For apoptosis assay, LE6 cells were incubated with indicated cell factors for 4 days, and then the cells were harvested and stained by FITC-labeled Annexin V/PI apoptosis assay kit (KeyGEN Biotech, China). The degree of apoptosis was tested by FACS. 3 independent assays were performed with at least 3 replicates.

### Western blotting and co-immunoprecipitation

After treatment with indicated cell factors, LE6 cells or LE6-sh*Smad4* cells were harvested and incubated in ice-cold RIPA lysis (Beyotime Institute of Biotechnology, China) plus protein inhibitor cocktail (Roche Ltd, Switzerland) for whole cell protein, and NE-PER Nuclear and Cytoplasmic Extraction Reagents (Thermo Fisher Scientific Inc, MA USA) for nuclear and cytoplasmic fractionation. Then the protein content was detected by BCA kit (Thermo Fisher Scientific Inc, MA USA). 60ug lysate was run on 15% or 10% PAGE polyacrylamide gel, transferred onto PVDF membranes (Roche Ltd, Switzerland). After blocked in 5% BSA in TBS at room temperature for 1 hour, the membranes were incubated with primary antibody at 4°C overnight. The details of primary antibodies were showen in Table 3. Then the membranes were washed with 0.1% tween-20 in TBS (TBST) and incubated with horseradish peroxidase-conjugated secondary or alkaline phosphatase antibody secondary antibody at 37°C for 1 hours (1:5000, Jackson Immuno Research Laboratories Inc, PA USA). Then the membranes were washed with TBST 3 times for 45 mins. Protein band immunoreactivity was revealed by chemiluminescence according to the manufacturer’s instructions (Thermo Fisher Scientific Inc, MA USA) and detected using an Alpha Innotech Fluorochem Imaging system (Alphatron Asia Pte Ltd, Singapore).

**Table 3:** Primary antibodies used for western-blot

**Primary antibodies**	**Dilusion**	**Manufacture**
Rabbite anti-p-samd2(Ser465/467)	1:1000	Cell Signaling Technology, USA
Rabbite anti-smad2	1:1000	Cell Signaling Technology, USA
Rabbite anti-p-samd3(ser423/425)	1:1000	Cell Signaling Technology, USA
Rabbite anti-smad3	1:1000	Cell Signaling Technology, USA
Rabbite anti-smad4	1:1000	Epitomics, China
Mouse anti-samd2/3		Santa Cruz Biotechnology Inc, USA
For WB	1:1000	
For IP	1:50	
Rabbite anti-p-ERK1/2(Thr202/Tyr204)	1:1000	Cell Signaling Technology, USA
Mouse anti-ERK1/2	1:2000	Cell Signaling Technology, USA
Rabbite anti-p-JNK1/2(Thr183/Tyr185)	1:1000	Cell Signaling Technology, USA
Rabbite anti-JNK1/2	1:1000	Cell Signaling Technology, USA
Rabbite anti-p-p38(Thr180/Tyr182)	1:1000	Cell Signaling Technology, USA
Rabbite anti-p38	1:1000	Cell Signaling Technology, USA
Rabbite anti-p-Rb(Ser807/811)	1:1000	Cell Signaling Technology, USA
Rabbite anti-Rb	1:1000	Santa Cruz Biotechnology Inc, USA
Rabbite anti-p21	1:1000	Epitomics, China
Rabbite anti-p15	1:1000	Epitomics, China
Rabbite anti-p27	1:1000	Cell Signaling Technology, USA
Rabbite anti-cyclinD1	1:5000	Epitomics, China
Mouse anti-cyclinA	1:500	Boster, China
Rabbite anti-cyclinE	1:500	Biolegend, USA
Rabbite anti-CDK2	1:500	Santa Cruz Biotechnology Inc, USA
Rabbite anti-CDK4	1:500	Santa Cruz Biotechnology Inc, USA
Mouse anti-GAPDH	1:10000	Santa Cruz Biotechnology Inc, USA
Mouse anti-β-actin	1:2000	Santa Cruz Biotechnology Inc, USA
Mouse anti-laminB	1:500	Proteintech Group, USA

For co-immunoprecipitation, LE6 cells or LE6-sh*Samd4* cells were serum-starved for 12 hours, and then treated with 200 ng/ml activin A for 1 hour. Cells were harvested and incubated with IP-lysis buffer (Beyotime Institute of Biotechnology, China). Cell lysis were incubated with mouse anti-*Smad2/3* polyclonal antibody (1:50, Santa Cruz Biotechnology, CA USA) at 4°C for 2 hours, followed by incubation with 20ul protein A/G agarose (Santa Cruz Biotechnology, CA USA) at 4°C overnight. Immunoprecipitates were washed 4 times with the lysis buffer and analysis by immunoblot using rat anti-*Smad4* monoclonal antibody (1:2000, Epitomics, CA USA).

### Statistical analyses

Data were expressed as mean values ± standard deviation. The related expression of genes was log transformed and variation with time assessed by ANOVA. The data of AnnexinV/PI assay, BrdU incorporation assay (both FACS and immunohistochemistry, except for Figure 5B) and liver/body weight ratio were analyzed by student’s t-test. ELISA, cell viability data and BrdU incorporation data (Figure 5B) were analyzed by unpaired student’s t-test or ANOVA by SPSS 11.5. A difference in P values of <0.05 was considered significant.

## Abbreviations

MAPK: Mitogen activated protein kinase; 2-AAF: 2-acetylaminofuorene; PH: Partial hepatectomy; TGF-β: Transforming growth factor β; ActR II A/B: Activin type II receptor A/B; ALK4: Activin A receptor-like kinase 4; CCK-8: Cell counting kit-8; R-smad: Receptor-regulated smad; co-smad: Common mediator Smad; pan-CK: Pan-cytokeratin; HPC: Hepatic progenitor cell; CDK: Cyclin-dependent kinase; HGF: Hepatocyte growth factor; EGF: Epithelial growth factor; BrdU: 5-bromo-2 –deoxyuridine; Ct: Cycle threshold.

## Competing interest

The authors confirm that there is no competing of interest.

## Authors’ contribution

LC: Did major experiments, animal model and acquisition of data, analysis and interpretation of data and drafted the manuscript. WZ: Participated in research design and animal experiment, acquisition of data and involved in drafting the manuscript. HfL: participated in research design, carried out immunohistochemistry assay, flow Cytometry assay, technique support, and helped to draft the manuscript. QdZ: Carried out the western blot assay, co-IP assay and helped to draft the manuscript. ZyD: Participated in cell culture, animal experiment and acquisition of some data. HqY: Participated in animal experiment, helped to analyses data. WbL: Participated in immunohistochemistry and acquisition of few data. YhW: Participated in western blot and IP assay and acquisition of few data. QM: Participated in lentivirus transfection and acquisition of few data. BxZ: Participated in research design and technique support. XpC: Study concept and design, analysis and interpretation of data, drafted the final manuscript, critical revision of manuscript for important intellectual content, technical support and study supervision. All authors read and approved the final manuscript.
